# Working on Cognitive Functions in a Fully Digitalized Multisensory Interactive Room: A New Approach for Intervention in Autism Spectrum Disorders

**DOI:** 10.3390/brainsci11111459

**Published:** 2021-11-03

**Authors:** Ilaria Basadonne, Melanie Cristofolini, Iris Mucchi, Francesco Recla, Arianna Bentenuto, Nadia Zanella

**Affiliations:** 1 Center for Mind/Brain Sciences (CIMeC), University of Trento, 38122 Trento, Italy; i.basadonne@unitn.it; 2 Laboratory of Observation, Diagnosis and Education (ODFLab), Department of Psychology and Cognitive Science, University of Trento, 38122 Trento, Italy; melanie.cristofolini@gmail.com (M.C.); iris.mucchi@gmail.com (I.M.); francesco.recla@gmail.com (F.R.); arianna.bentenuto@unitn.it (A.B.)

**Keywords:** autism spectrum disorders (ASD), multisensory interactive rooms, patient–therapist interaction, cognitive functions, sensory atypicalities

## Abstract

The feasibility of working on cognitive functions with children and adults with Autism Spectrum Disorders (ASD) inside Multisensory Interactive Rooms (MIRs) has been poorly investigated, even if sensory atypicalities are common in ASD and usual intervention rooms could represent a challenging sensory setting for patients with ASD. We hypothesized that the possibility to calibrate the sensory stimulation offered by this type of environment, able to promote a positive emotional state in patients with ASD, can consequently favor the interaction with the therapist and the motivation towards activities targeting cognitive functions. High- and low-functioning children and low-functioning adolescents/adults underwent five sessions in a fully digitalized MIR, working on sustained attention, selective attention, association, single inhibition, receptive communication, verbalization, and turn. We developed specific protocols calibrated for sensory stimulation and difficulty level based on the characteristics of the participants. We found statistically significant improvements in all functions, except association, in the children’s group. Therefore, a fully digitalized MIR seems suitable for intervention on cognitive functions in ASDs, but further investigations are needed to better address possible differences related to age and functioning level.

## 1. Introduction

Autism Spectrum Disorder (ASD) is an early-onset neurodevelopmental condition characterized by persistent difficulties in social communication and interaction and repetitive and restricted behaviors and interests [1]. More than 90% of people with ASD show sensory atypicalities [2]. Nevertheless, only since The Diagnostic and Statistical Manual of Mental Disorders- Fifth Edition (DSM–5) were hyper- or hyporeactivity to sensory input and unusual interest in sensory aspects of the environment inserted as a diagnostic criterion for ASD [1]. This has given even greater awareness of the importance of considering sensory aspects in the clinical picture of ASD.

So far, several studies have shown that sensory challenges in ASD affect all sensory channels [2]. It seems that, in particular, atypical sensory responses to smell and taste allow differentiation between children with ASD and others with different neurodevelopmental disorders [3]. However, discordant results have been reported on the actual differences in the sense of smell between ASD and controls. Indeed, Galle showed that ASD adults have the same ability to detect and discriminate odors as do controls but a lower ability to identify them [4], while Ashwin showed a greater ability to detect odors, positively correlated with the severity of autistic traits [5]. In children with ASD, a lower ability to identify odors was found compared to controls [6] or the same identification ability but a lower detection ability [7]. To overcome the possible effect of different language skills on the performance in these kinds of studies, Rosenkrantz measured physiological responses to odors in ASD children and controls, finding ASD children sniffing equally in the case of pleasant and unpleasant odors, even if no differences in odor perception were reported. In addition, altered sniffing responses correlated positively with ASD severity [8]. As for taste, less accuracy was found in identifying acid and bitterness in adolescents with ASD [6] and also sweetness in adults with ASD [9]. Conflicting results have also been reported for touch. In fact, Blackmore [10] found an impairment in detecting tactile stimuli in adults and children with ASD while no difference to controls was detected in other studies [11,12]. Interestingly, Coskun [13] found a greater distance in the cortical representation of thumb and lips in ASD. As for hearing, children with ASD show difficulty in discriminating two sounds when presented close together [14] and greater latency in the neural response during magnetoencephalography both for pure tones and for complex social stimuli (e.g., speech), predictive of the severity of ASD traits [15]. Moreover, a superior ability was found to discriminate between two equal and different tones and categorize them as higher/lower [16]. Finally, sight seems to be characterized by a greater orientation to details and the preference for parts of the scene characterized by contrast or color (pixel level) rather than size, density, or outline of objects (object level) or linked to parts of a text, tools, faces (semantic level) [17]. Furthermore, atypical processing of moving dots was detected [18].

The conflicting results obtained so far are probably partly attributable to the different stimulation used in the various studies and partly to the intrinsic heterogeneity of the samples involved, a consequence of the phenotypic variability in terms of traits and severity that characterizes ASD. Nonetheless, according to Bogdashina [19] and Grandin [20], people with ASD perceive the entire scene to which they are exposed as a single entity with all its details, without being able to break it down into meaningful units (e.g., objects/people) in relation to each other. They then elaborate the details of the scene, which each time attract their attention, but without being able to give an interpretation to the whole scene. They also show great difficulty in filtering the non-salient elements, so the simultaneous processing of all the stimuli becomes impossible and overloading, requiring a lot of time and energy and inducing a high level of stress. Furthermore, even the slightest changes in the scene make it different and no longer recognizable, generating fear and frustration. All this would also cause resistance to changes and difficulty in adapting to new environments. Moreover, Delacato [21] classifies the sensory channels in ASD as *iper*, meaning too open, that is, too much information enters for the brain to manage, or *ipo*, meaning not sufficiently open, that is, too little information enters. Consequences of the first condition are disturbance/pain or fascination for certain stimuli (different from person to person). Instead, *ipo* channels lead people to appear absent or to seek sensory stimulation to activate the flow of information. In addition, Ayres [22] recognizes in ASD a difficulty in sensory integration, that is, the neurobiological process that integrates and organizes all the sensations that come both from the external environment and from one’s own body through the various sensory channels. Moreover, other conditions that often occur in comorbidity with ASD, such as, e.g., attention-deficit hyperactivity disorder (ADHD), can further complicate the sensory challenges in ASD. Interestingly, Dellapiazza found that children with ASD and ADHD showed more atypical sensory processing than children with only a diagnosis of ASD [23]. Considering these sensory peculiarities, it is understandable how everyday environments can be challenging for people with ASD.

Furthermore, social interaction can also be affected by sensory atypicalities. In fact, some studies have highlighted how greater social difficulties are associated with profiles of hyper- or hypo-responsiveness to sensory stimuli in ASD [24,25,26]. In particular, Corbett found that children with ASD showed higher stress levels, assessed measuring saliva cortisol, in response to the Peer Interaction Paradigm, a protocol that simulates social interaction in an ecological context if compared to typically developing children. Interestingly, higher cortisol levels were associated with higher sensory sensitivity [27].

Therefore, it can be hypothesized that environments in which sensory stimulation can be calibrated on the characteristics of the person with ASD can favor their well-being and functioning, both cognitive and social. In the 1970s, Hulsegge and Verheul, noting the positive effect of exposure to sensory stimuli in people with intellectual disabilities, set up environments specifically dedicated to sensory stimulation. Inside, there was a soft corner with cushions and hay, hidden sound toys, light projections on the ceiling, an area with musical instruments, headphones and speakers, tubs with sand, tactile objects, a fan blowing on scraps of paper, and an area for olfactory stimulation with perfumes, soaps, herbs, and trays with foods of different flavors. Hulsegge and Verheul developed a way of using the multisensory environment called Snoezelen, from the Dutch words “snuffelen” (=explore) and “doezelen” (=relax), based on free exploration of the environment by the user. It aimed to promote well-being and activation through the selected sensory stimuli and favor the relationship with the operator, mediated by the shared sensory experience [28]. Over the years, the elements present inside the Snoezelen rooms have been gradually modernized and, alongside the physical objects, digital, sometimes interactive, light projections have also been introduced. The use of multisensory rooms has been conducted over time mainly following the Snoezelen approach and involving users with intellectual disabilities [29], but only a few studies have reported experiences with people with ASD so far. In particular, Fava and Strauss found that Snoezelen intervention reduced disruptive behaviors in adults with ASD and intellectual disability [30]. In addition, Kaplan showed behavioral improvements during Snoezelen occupational therapy in two adults with ASD and intellectual disabilities, a higher engagement during a functional task immediately following the treatment sessions, and a reduction in challenging behavior even on the following days [31]. Moreover, Smet reported that parents of preschool-aged children with mild to severe ASD noticed a more positive emotional state in their children during sessions in a multisensory room compared with the home environment [32]. Interestingly, Novakovic found that three sessions a week in the Snoezelen room for 3 months had effects on reducing the severity of ASD and repetitive and stereotyped behaviors in adolescents and adults [33]. Furthermore, the strong link between sensory aspects and other ASD traits was also highlighted by Woo and Leon. They reported that children with ASD undergoing sensorimotor enrichment at home for 6 months showed an improvement in ASD severity and in cognition compared with an ASD control group receiving only standard care [34].

### Aims and Hypothesis

Alongside sensory atypicalities and social communication and interaction issues, people with ASD often show difficulties in other cognitive functions, such as attention and executive functions [35]. These aspects are generally addressed in the therapeutic intervention in traditional settings. Nevertheless, even usual intervention rooms could represent a challenging sensory setting for patients with ASD.

Thus, this work aimed to assess if the activities provided by a fully digitalized multisensory interactive room not only favor the well-being, but are also suitable to work on cognitive functions with children, adolescents, and adults with ASD and different functioning levels. To our knowledge, no studies of this type have been conducted so far.

We hypothesized that the possibility of calibrating the sensory stimulation offered by this type of environment and the sense of control on the environment experienced in the interaction with the stimuli, both able to promote a positive emotional state in the patients with ASD, can consequently favor the interaction with the therapist and the engagement towards activities, promoting the achievement of the intervention goals.

## 2. Materials and Methods

### 2.1. Participants

For this study, *n* = 8 adolescents/adults (three females and five males) aged 16–36 years (mean = 24.8, SD = 7.01) and 13 children (ten males and three females) aged 3–8 years (mean = 5.57, SD = 1.11) were enrolled. The adolescents/adults were recruited from the guests of “Casa Sebastiano”, a forefront residential center for people with ASD located in Coredo, in the Province of Trento, in North Italy. The children were recruited from the participants in “Terapia in Vacanza”, a 1-week, daytime, intensive intervention camp (Monday–Friday) for children that takes place at “Casa Sebastiano” in the summer. It is organized by the Laboratory of Observation, Diagnosis, and Education (ODFLab) of the University of Trento, a clinical research center specialized in neurodevelopmental disorders.

All participants had previously received by licensed independent clinical psychologists a diagnosis of ASD according to the DSM-5 and the Autism Diagnostic Observation Schedule—Second edition (ADOS-2), a gold standard instrument for ASD diagnosis [36]. Moreover, all participants had also undergone a cognitive assessment within a year before the enrollment, according to which all adolescents and adults were found to have low cognitive functioning (Intelligence quotient < 70), while, among the children, nine were high-functioning (Intelligence quotient ≥ 70) and four were low-functioning. All participants were able to verbalize, at least single words.

Informed consent was obtained from all parents of the participants involved in the study.

### 2.2. The Multisensory Interactive Room

**Figure 1:**
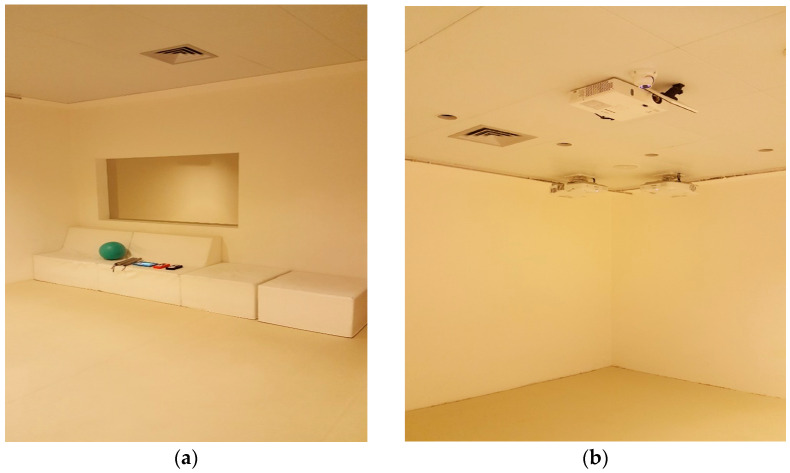
(**a**) Interior of the Multisensory Interactive Room, sofa and one-way mirror; (**b**) Interior of the Multisensory Interactive Room, projectors.

The Multisensory Interactive Room (MIR) used in this study is located inside “Casa Sebastiano”. Produced by the company Omi OM Interactive Ltd. (Hemel Hempstead-UK), it has an area of 30 m^2^ with three completely white walls, while on the fourth one, there is the entrance door and a one-way mirror. The floor is white and soft. On the same side of the door, a sofa allows one to sit and relax. This is the only physical item inside the room (Figure 1a). On the ceiling, also white, are installed a central projector and six peripheral projectors that reproduce images on the floor and on the walls with which it is possible to interact (Figure 1b).

**Figure 2:**
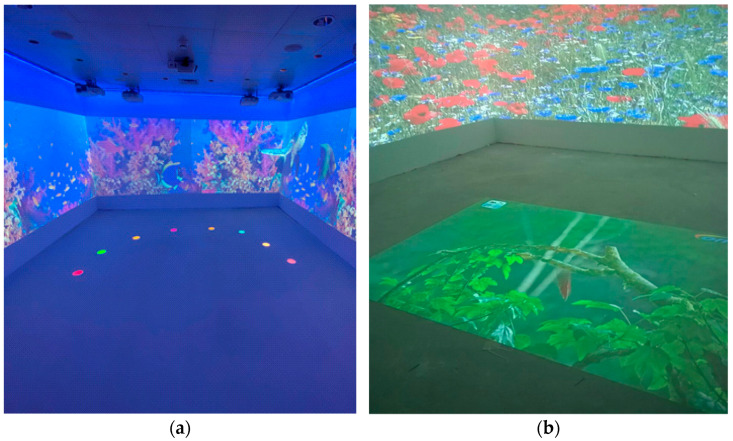
(**a**) Interior of the Multisensory Interactive Room, eight light beams projected on the floor. (**b**) Interior of the Multisensory Interactive Room, interactive area on the floor.

The interaction on the walls occurs thanks to the interruption, using special soft rods, of eight beams of light projected in a semicircle around the interactive floor area to make the visual and sound stimuli appear and disappear on the walls (Figure 2a).

The interaction with the projections on the floor (Figure 2b) is possible through simple movements of the hands, feet, or objects. The MIR software offers about 350 different projections whose features can be modified according to the characteristics of the individual users. Six types of interaction with the floor are provided, and each projection allows only one kind of interaction:

QUIZ: Simple questions to be answered by touching the correct alternative and obtaining visual and sound feedback. The number of alternatives can be increased up to four.

SCATTER: Various elements are projected onto the floor, partially overlapping each other. Passing over them, they begin to move, and an image connected to them is discovered. After a lapse of time, which can be changed, the elements close again, recreating the starting situation. Elements and underlying images can be modified.

SPLAT: Single moving objects are projected onto the floor. When squeezed, they reveal a new image. In some activities, pressing all the elements results in a final visual and sound feedback. The type of elements, their number, size, speed, and the associated images can be modified.

WATER: Landscapes with moving water surfaces (e.g., a lake) are projected onto the floor. By passing over it, the user causes the intensification of the motion and the activation of sound stimuli such as the lapping of the waves and other sounds of nature.

WIPE: As the user passes over the projected static image, it turns color or disappears, gradually revealing a new image and activating sound stimuli. After a period of time, which can be set, the initial image reappears. The images can be modified.

ZONES: Single static stimuli are projected on the floor (up to a maximum of eight); by pressing them, they animate or other associated elements appear together with a corresponding sound. 

All projections are accompanied by sound stimuli (background music and/or specific sounds associated with a particular action of the user).

### 2.3. Procedure

For this study, we decided to test the possibility of working inside the MIR on the following functions: *sustained attention* (SA), in our case, the ability to maintain an instruction until the complete execution of the task; *selective attention* (SeA), i.e., the ability to focus on one or more elements while ignoring distractors; *association* (A), i.e., the ability to combine two actions, in our case, performing an action while looking at the targets of the action; *single inhibition* (SI), i.e., the ability to refrain from a behavior, e.g., implemented previously, in favor of another required behavior.

Furthermore, we decided to work on aspects directly linked to the relationship with the therapist, namely, *receptive communication* (RC), i.e., the ability to understand instructions; *verbalization* (V), i.e., the ability to produce words or sentences at the request of the therapist; *turn* (T), i.e., the ability to consider the presence of the other and to recognize and respect their role in the interaction.

Considering the 350 different projections offered by the software, we selected a set of them that we considered functional to work on the functions described above. We then developed a set of activities to be completed using those projections. We tested these activities in a pilot phase during the 2 months before the start of this study, in which we involved two high-functioning children and two low-functioning adults. In this way, we reduced the number of selected projections and activities to the most effective ones.

Subsequently, we developed two different protocols, one for the high functioning and one for the low functioning participants, containing the activities to be carried out during each session in the MIR. For the low-functioning participants, we selected activities that are easier to be performed and with a lower sensory stimulation. The protocols were constructed so that each activity (e.g., hitting the moving objects in turn) was declined in a set of tasks, each of which corresponded to a function under study (e.g., looking at the object while hitting it = *association*). Moreover, each function was worked on in at least two different activities and each activity was repeated twice (Table A1 and Table A2).

For the group of children, five sessions of 30 min each were carried out in the MIR, one for each day of stay at the summer camp. For the adolescent and adult group, as some participants were at “Casa Sebastiano” only once a week, we decided to expose them to one session of 30 min per week in the MIR for 5 weeks.

In all sessions in the MIR, the tasks carried out were the same for each protocol. The sessions in the room were conducted for each participant always at the same time during the day to reduce the variability linked to factors such as fatigue or differences in the activities carried out before the sessions in the room.

### 2.4. Measures

To evaluate possible improvements in the functions under study, we developed two observation forms, for the high- and the low-functioning participants, respectively. In each form, all selected activities were listed, together with their associated tasks and functions. A 5-point Likert scale was used for each task (and function) evaluation. The scoring levels reflected the degree of help needed by the participant to carry out the task, as follows:

- 1 = the task was not completed
- 2 = the task was completed with a physical prompt
- 3 = the task was completed with a verbal prompt
- 4 = the task was completed almost autonomously
- 5 = the task was completed autonomously

Moreover, the associated function was listed beside each task, and the same score obtained in the task was also assigned to the function. Each task was performed twice and consequently evaluated twice on the observation form. Therefore, each function associated with that task was also evaluated twice. As mentioned above, the protocols were structured so that each participant could work on each function at least in two tasks.

The performance evaluation took place in the first and fifth sessions. In each session, the average score obtained by the participant for each function was calculated.

### 2.5. Statistical Analysis

Considering the different ages and frequency of sessions in the MIR (daily vs. weekly), we decided to separately analyze the performance of the children’s and adolescents/adults’ group.

Using a repeated measures MANOVA, we assessed statistically significant differences in the scores obtained between session 1 (S1, considered as a baseline) and session 5 (S5) in each of the two groups, with the cognitive functions under study as multiple dependent variables. We then tested each function independently using a Wilcoxon Signed-rank test on repeated measures (with Bonferroni correction for multiple comparisons).

Moreover, increments were then calculated for each function between S1 and S5 to highlight those in which there was the greatest and the smallest increase in each group.

Only in the children’s group, a mixed-effect ANOVA was conducted to investigate a possible combined effect of intervention and functioning level.

All analyses were conducted using the R software.

## 3. Results

### 3.1. Children

Looking at the performances in S1 and S5 from a descriptive point of view, children scored averagely at the lowest in *verbalization* (2.83) and at the highest in *association* (4.62) in S1, whereas in S5 the lowest average performance was in *turn* (4.20) and the highest again in *association* (4.86), as shown in Table 1.

Interestingly, some children received the maximum score (5 points) in S1 in *sustained attention* (3), *association* (7), *receptive communication* (2), and *verbalization* (1). 

Nevertheless, in S5, the number of children reaching the maximum score increased: *sustained attention* (3), *selective attention* (4), *association* (10), *single inhibition* (4), *receptive communication* (3), and *verbalization* (6). 

Repeated measures MANOVA highlighted a significant effect of time (*p* < 0.001). Wilcoxon Signed-rank test on repeated measures and one-sided (with Bonferroni correction) showed a statistically significant increase in performance between S1 and S5 in six functions: *sustained attention* (*p* < 0.05), *selective attention* (*p* < 0.01), *single inhibition* (*p* < 0.01), *receptive communication* (*p* < 0.05), *verbalization* (*p* < 0.01), and *turn* (*p* < 0.01).

After increments’ calculation between S1 and S5 for all children in each function, we found the most increase in *verbalization* (1.56), with the lowest increase in *association* (0.24).

Additionally, we also assessed possible differences between high- (HF) and low-functioning (LF) children. 

However, in S1, HF and LF children scored averagely at the highest in *association* (4.85 and 4.11), and at the lowest in *verbalization* (2.56) and *single inhibition* (2.31), respectively. Additionally, in S5, the HF and the LF groups scored averagely at the highest in *association* (5 and 4.55), and at the lowest in *turn* (4.34) and *selective attention* (3.31), respectively (Table 2). 

After increments’ calculation between S1 and S5, both HF and LF children showed the lowest average increase in *association* (0.15 and 0.43, respectively), and the highest in *verbalization* (1.96) for the HF group and in *single inhibition* (1.23) for the LF group. 

To explore a possible combined effect of intervention and children’s level of functioning, we conducted a mixed-effect ANOVA for each of the functions.

**Figure 3:**
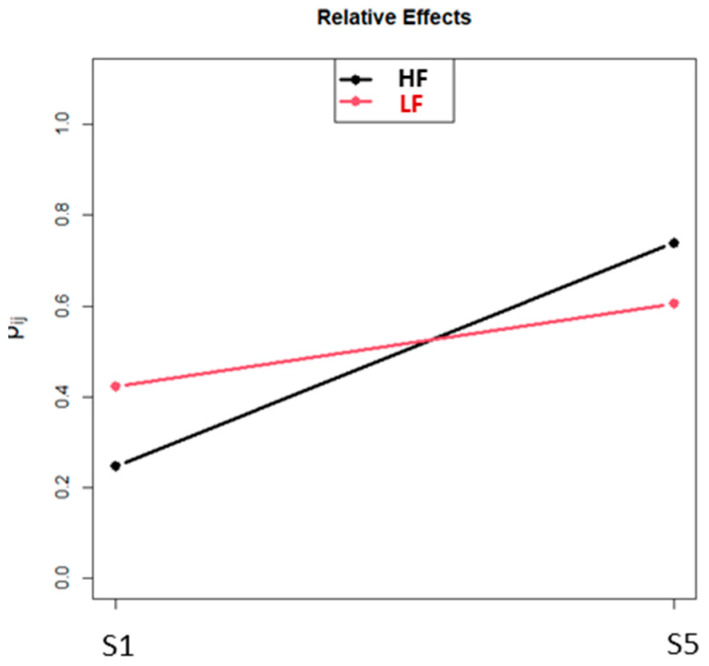
Mixed-effect ANOVA considering differences in performances between S1 and S5 and functioning level (HF vs. LF) in the children’s group.

The only function that showed a significant interaction was *verbalization* (*p* = 0.0009) (Figure 3).

### 3.2. Adolescents/Adults

Adolescents’/adults’ performance scored averagely at the lowest in *turn*, both in S1 (2.76) and S5 (3.63), and at the highest in *association*, again both in S1 (4.36) and S5 (4.75), as shown in Table 1.

Few adolescents/adults received the maximum score (5 points) in S1 in the functions *selective attention* (1), *association* (2), *turn* (2), and *verbalization* (3), whereas, in S5, the number of participants reaching the maximum increased: *selective attention* (2), *association* (4), *turn* (4), and *verbalization* (5).

After increments’ calculation for all adolescents/adults between S1 and S5 in each function, we found the most increase in *verbalization* (1), while the lowest increase was obtained in *selective attention* (0.24).

Although the adolescents/adults’ group showed an increase in all functions, repeated measures MANOVA for differences in performances between S1 and S5 with all the cognitive functions under study as multiple dependent variables did not show any significant effect of time. In an attempt to overcome the possible negative impact of the low sample size on the possibility to detect an effect using MANOVA, we also analyzed the cognitive functions individually, running a Wilcoxon Signed-rank test on repeated measures and one-sided (with Bonferroni correction). Even in this case, we did not find any statistically significant increase in performance between S1 and S5. 

## 4. Discussion

Thanks to the interaction between our sense organs, what we perceive, and the central nervous system, we are able to interpret and understand the world around us [37]. However, in people with Autism Spectrum Disorder, the real and perceptive worlds differ and the brain struggles to give real meaning to what is perceived [21]. Therefore, common perceptual experiences for typically developing individuals can be highly disturbing for people with ASD. Furthermore, optimal levels of stimulation appear to vary from person to person [38].

The few studies conducted in the past on the use of multisensory rooms with people with ASD highlighted a positive emotional state of the users inside the room and a reduction in disruptive behaviors [30,31,32,33]. Moreover, a sensorimotor enrichment intervention in domestic contexts was found to improve ASD severity and cognition in children with ASD [34].

The novelty of our study was to investigate whether a fully digitized multisensory room is suitable for working with people of different ages and cognitive profiles and also on some cognitive functions that are often compromised in ASD, such as *sustained* and *selective attention, association* (in our case performing an action while looking at the targets of the action), *single inhibition* (i.e., the ability to refrain from a behavior, e.g., implemented previously, in favor of another required behavior), *receptive communication*, *verbalization,* and *turn.* Considering the functions linked to attention (*sustained* and *selective attention*) and executive functions (*single inhibition*), we found that most participants scored under the maximum in S1. This indicates that the selected activities were able to bring out the typical difficulties of people with ASD in these functions, as reported in the literature. In fact, attention disorders seem central in ASD, particularly for divided attention tasks, where people with ASD show longer reaction times and difficulties in detaching attention from a task, especially if incongruent [39]. Moreover, as for executive functions, a tendency to persevere and the inability to develop alternative strategies has been reported [40,41,42].

Even regarding the functions related to the interaction with the therapist (*receptive communication, verbalization,* and *turn*), the selected activities effectively highlighted participants’ difficulties. In particular, children showed the lowest average score in *verbalization* and adolescents/adults in *turn*. This is understandable considering the well-known difficulties in intersubjectivity that characterize ASD [43,44,45,46] and the challenges in receptive communication, at various levels, even in verbal persons. In fact, there is a difficulty in recognizing and differentiating words from other sounds and in identifying the beginning and the end of the words [47]. Regarding verbalization, people with ASD can be slow to start talking or may not learn to speak at all; others may learn to produce words and sentences but have difficulty using them effectively to accomplish social goals [48].

However, some children and adults scored the maximum in S1. This can be attributed to the particular characteristics of each participant. In fact, ASD traits occur along a continuum of severity. Thus, the selected activities may have been too easy to perform per se. On the other hand, the particular sensory setting inside the MIR may have facilitated the participants’ performing those activities. For example, in the case of *association,* more than half of the children and two adults received the maximum score in S1. This could be attributed to the subjects’ particular interest in the digital and interactive projections offered by the MIR and to the absence of distractors (e.g., noise from outside, light through windows, etc.) that rendered it easier to maintain the association between the two actions.

In our study, we also compared the performance between S1 and S5 in the children’s and adolescents/adults’ group. As for the children, we obtained statistically significant increases in all functions except association. The highest increase was in *verbalization*. Therefore, we can infer that the selected activities were challenging enough to stimulate an improvement but not too difficult to induce participants to desist from performing them. Moreover, previous studies showed that MIRs favor the well-being of the users [30,31,32,33]. Thus, we can hypothesize that the positive emotional state while performing the tasks facilitated the children’s improvements, particularly in the functions related to the interaction with the therapist, such as *verbalization*. Furthermore, the visual and sound stimulations provided by the MIR and the possibility of interacting with them may have engaged the participants and increased their motivation towards the tasks and to communicate with the therapist, favoring improvements even after a few sessions. Notably, some children reached the maximum score in S5. If the intervention in the room had continued beyond the five sessions under study, we could have proposed even more demanding activities to this group. This is possible thanks to the great flexibility of the MIR, which allows the activities to be modified based on the characteristics of the users.

Interestingly, we found a combined effect of the intervention in the MIR and participants’ cognitive functioning only in *verbalization*. The HF children had, in S1, an average score a little lower than the LF ones, but obtained a higher score in S5. It is possible that precisely in the HF group, the positive effect of MIR on the mood was more remarkable because of their greater awareness of what surrounds them, and this aspect, together with enthusiasm for the proposed activities, increased their motivation to communicate.

Regarding the adolescents/adults’ group, even if we found increases in all functions between S1 and S5, they were not statistically significant. However, this could be attributable to the small sample size, which allowed only a large effect size to be detected. The function in which they obtained the lowest average score, in S1 and S5, was *turn*. This is understandable, given that it requires high cognitive skills. Nevertheless, adolescents/adults also showed the highest increase in *verbalization*, perhaps for the same reasons as for the children.

Our study has some limitations: first, the small sample size and its composition, with only four low-functioning children and the lack of high-functioning adolescents/adults. This affects the generalizability of our results. Moreover, the frequency of the sessions in the MIR was different for children and adolescents/adults due to the structuring of the “Terapia in Vacanza” camp and the weekly activities at “Casa Sebastiano”. Thus, we did not have the possibility to dissociate this additional variable from the age variable. Another limitation is the lack of a control group. Since the number of participants we could get access to was limited, we preferred to involve all participants in the activities in the MIR. In this way, however, it was not possible to assess whether the improvements we detected were actually attributable to the activities carried out during the five sessions in the MIR, as we hypothesized. Furthermore, we did not have the opportunity to test the protocol tasks with each participant before starting the study to find the exact baseline in each function. This led to the achievement of the maximum score in the first session by some participants.

## 5. Conclusions

In this study, we tested whether a fully digitalized MIR is suitable for working on cognitive functions in children, adolescents, and adults with ASD and different functioning levels. Even if the results of our study are to be considered exploratory, they represent an interesting indication of the high therapeutic potential of the MIR. In fact, the possibility of calibrating the activities for sensory stimulation and difficulty level on the participants’ characteristics seems to make the MIR an effective intervention setting, able to favor improvements in cognitive functions in ASD, likely even after few sessions. However, further research with a larger sample size is needed, also to address better possible differences related to age, functioning levels, and intervention frequency. In addition, considering comorbidities characterized by atypical sensory processing, such as ADHD, will allow a more articulated framing of the results of studies on MIRs. Moreover, it would be interesting to evaluate whether the intervention on cognitive functions in MIR is more effective compared with that in the usual therapeutic setting and if the improvements achieved in the MIR are generalizable to everyday life.

## Figures and Tables

**Table 1:** Children’s and adolescents’/adults’ performances in S1 and S5.

Functions	Sessions	Children		Adolescents/Adults	
		Mean (SD)	Min/Max (^2^)	Mean (SD)	Min/Max (^2^)
SA ^1^	S1	3.73 (0.83)	2.5/5 (3)	3.76 (0.82)	2.27/4.71
	S5	4.53 (0.55)	3.36/5 (3)	4.36 (0.35)	3.73/4.85
SeA ^1^	S1	3.01 (1.05)	1.00/4.25	3.66 (1.52)	1.00/5 (1)
	S5	4.34 (1.04)	1.75/5 (4)	3.93 (1.37)	1.00/5 (2)
A ^1^	S1	**4.62** **(0.65)**	2.91/5 (7)	**4.36** **(0.93)**	2.73/5 (2)
	S5	**4.86** **(0.34)**	3.82/5 (10)	**4.75** **(0.31)**	4.27/5 (4)
SI ^1^	S1	3.30 (0.98)	1.33/4.71	3.30 (1.24)	1.00/4.46
	S5	4.45 (0.81)	2.83/5 (4)	3.68 (1.21)	1.00/4.77
RC ^1^	S1	3.85 (0.75)	2.55/5 (2)	3.89 (0.67)	2.73/4.75
	S5	4.53 (0.58)	3.27/5 (3)	4.37 (0.93)	3.87/4.78
V ^1^	S1	**2.83** **(1.19)**	1.00/5 (1)	3.38 (1.60)	1. 00/5 (3)
	S5	4.39 (0.67)	3.21/5 (6)	4.38 (0.92)	3.00/5 (5)
T ^1^	S1	3.08 (0.93)	1.00/4.50	**2.75** **(1.67)**	1.00/5 (2)
	S5	**4.20** **(0.51)**	3.17/4.83	**3.63** **(1.77)**	1.00/5 (4)

^1^ SA (sustained attention); SeA (selective attention); A (association); SI (single inhibition); RC (receptive communication); V (verbalization); T (turn). (^2^) Number of children who scored the maximum for the function in the session.

**Table 2:** HF and LF children’s performances in S1 and S5.

Functions	Sessions	High-Functioning		Low-Functioning	
		Mean (SD)	Min/Max (^2^)	Mean (SD)	Min/Max (^2^)
SA ^1^	S1	3.95 (0.92)	2.25/5 (3)	3.22 (0.20)	3.00/3.44
	S5	4.81 (0.20)	3.36/5 (3)	3.90 (0.60)	3.36/4.70
SeA ^1^	S1	3.28 (1.00)	1.00/4.25	2.41 (1.03)	1.00/3.33
	S5	4.80 (0.21)	4.44/5 (3)	**3.31** **(1.46)**	1.75/5 (1)
A ^1^	S1	**4.85** **(0.21)**	4.20/5 (6)	**4.11** **(0.98)**	2.91/5 (1)
	S5	**5** **(0.00)**	5/5 (9)	**4.55** **(0.52)**	3.82/5 (1)
SI ^1^	S1	3.75 (0.67)	2.50/4.71	**2.31** **(0.87)**	1.33/3.40
	S5	4.86 (0.13)	4.67/5 (3)	3.54 (1.00)	2.83/5 (1)
RC ^1^	S1	4.02 (0.80)	2.55/5 (2)	3.43 (0.54)	3.00/4.25
	S5	4.80 (0.21)	4.40/5 (3)	3.93 (0.70)	3.27/4.88
V ^1^	S1	**2.56** **(1.34)**	1.00/5 (1)	3.45 (0.37)	3.20/4.00
	S5	4.52 (0.63)	3.50/5 (5)	4.10 (0.77)	3.21/5 (1)
T ^1^	S1	2.95 (1.09)	1.00/4.50	3.37 (0.43)	3.00/3.80
	S5	**4.34** **(0.35)**	3.67/4.75	3.88 (0.72)	3.17/4.83

^1^ SA (sustained attention); SeA (selective attention); A (association); SI (single inhibition); RC (receptive communication); V (verbalization); T (turn). (^2^) Number of children who scored the maximum for the function in the session.

## Data Availability

The data supporting the findings reported in this study are not publicly available due to privacy reasons. Nevertheless, they can be requested from the corresponding author for well-motivated reasons.

## Appendix A

In Appendix A are reported the protocols that were used for the high-functioning (Table A1) and low-functioning participants (Table A2). For each activity, the type of interaction with the MIR and the single tasks are listed, together with the related cognitive functions. Each activity was repeated twice.

**Table A1:** Protocol used with the high-functioning participants.

Type of Interaction	Activity	Tasks	Functions ^1^
SPLAT	Crossing the floor area without touching any moving element	Understanding the instruction	RC
		Looking at the elements while crossing the area	A
		Not touching the elements	SI
		Maintaining the instruction	SA
	Following the chosen element without hitting it until the therapist says “go”	Understanding the instruction	RC
		Looking at the chosen element	SeA
		Not hitting the element	SI
		Maintaining the instruction	SA
	Hitting only the chosen type of elements	Understanding the instruction	RC
		Looking at the chosen elements while hitting	SeA
		Hitting only the chosen elements	SI
		Maintaining the instruction	SA
	Hitting the other types of elements	Understanding the instruction	RC
		Looking at the chosen elements while hitting	SeA
		Hitting only the chosen elements	SI
		Maintaining the instruction	SA
	Hitting the elements in turn with the therapist	Understanding the instruction	RC
		Looking at the elements while hitting	A
		Keeping the turn	T
WALLS	Activating the elements on the walls	Understanding the instruction	RC
		Looking at the elements while activating	A
		Describing the elements	V
		Maintaining the instruction	SA
	Activating the elements on the walls in turn with the therapist	Understanding the instruction	RC
		Looking at the elements while activating	A
		Keeping the turn	T
QUIZ	Questions with answers to choose between	Understanding the instruction	RC
		Describing the alternatives	V
		Maintaining the instruction	SA

^1^ SA (sustained attention); SeA (selective attention); A (association); SI (single inhibition); RC (receptive communication); V (verbalization); T (turn).

**Table A2:** Protocol used with the low-functioning participants.

Type of Interaction	Activity	Tasks	Functions ^1^
WIPE	Coloring the element before it becomes black	Understanding the instruction	RC
		Looking at the element while coloring	A
		Naming the element	V
		Maintaining the instruction	SA
SPLAT	Hitting all moving elements	Understanding the instruction	RC
		Looking at the single elements	SeA
		Maintaining the instruction	SA
		Maintaining the instruction	SA
	Hitting only the chosen type of elements	Understanding the instruction	RC
		Looking at the elements while hitting	SeA
		Hitting only the chosen elements	SI
		Maintaining the instruction	SA
	Hitting only the other types of elements	Understanding the instruction	RC
		Looking at the elements while hitting	SeA
		Hitting only the chosen elements	SI
		Maintaining the instruction	SA
SCATTER	Passing the ball to the therapist and back, the ball rolls on the floor	Understanding the instruction	RC
		Keeping the turn	T
ZONES	Touching the elements in turn with the therapist	Understanding the instruction	RC
		Looking at the elements while touching	A
		Naming the elements	V
		Keeping the turn	T
QUIZ	Simple questions with two alternative answers to choose between	Understanding the instruction	RC
		Naming the alternatives	V
		Maintaining the instruction	SA

^1^ SA (sustained attention); SeA (selective attention); A (association); SI (single inhibition); RC (receptive communication); V (verbalization); T (turn).
